# Wild potato ancestors as potential sources of resistance to the aphid *Myzus persicae*

**DOI:** 10.1002/ps.6957

**Published:** 2022-05-21

**Authors:** Jamin Ali, Islam S Sobhy, Toby JA Bruce

**Affiliations:** ^1^ School of Life Sciences Keele University Keele UK; ^2^ Department of Plant Protection, Faculty of Agriculture Suez Canal University Ismailia Egypt; ^3^ Present address: School of Biosciences Cardiff University Cardiff CF10 3AX UK

**Keywords:** wild potato, aphids, parasitoid, *Solanum* spp., plant resistance, pest management

## Abstract

**BACKGROUND:**

Plant resistance to insects can be reduced by crop domestication which means their wild ancestors could provide novel sources of resistance. Thus, crossing wild ancestors with domesticated crops can potentially enhance their resistance against insects. However, a prerequisite for this is identification of sources of resistance. Here, we investigated the response of three wild potato (*Solanum stoloniferum* Schltdl.) accessions and cultivated potato (*Solanum tuberosum)* to aphid (*Myzus persicae* Sulzer) herbivory.

**RESULTS:**

Results revealed that there was a significant reduction in aphid survival and reproduction on wild potato accessions (CGN18333, CGN22718, CGN23072) compared to cultivated (Desiree) potato plants. A similar trend was observed in olfactometer bioassay; the wild accessions had a repellent effect on adult aphids. In contrast, among the tested wild potato accessions, the parasitoid *Diaeretiella rapae* (M'Intosh) was significantly attracted to volatiles from CGN18333. Volatile analysis showed that wild accessions emitted significantly more volatiles compared to cultivated potato. Principal component analysis (PCA) of volatile data revealed that the volatile profiles of wild and cultivated potato are dissimilar. β‐Bisabolene, (*E*)‐β‐farnesene, *trans*‐α‐bergamotene, d‐limonene, (*E,E*)‐4,8,12‐trimethyl‐1,3,7,11‐tridecatetraene (TMTT), and *p*‐cymen‐7‐ol were the main volatiles contributing to the emitted blends, suggesting possible involvement in the behavioural response of both *M. persicae* and *D. rapae*.

**CONCLUSION:**

Our findings show that the tested wild accessions have the potential to be used to breed aphid‐resistant potatoes. This opens new opportunities to reduce the aphid damage and to enhance the recruitment of natural enemies. © 2022 The Authors. *Pest Management Science* published by John Wiley & Sons Ltd on behalf of Society of Chemical Industry.

## Graphical abstract

Our study found evidence of aphid resistance in wild potato accessions. Aphid performance was significantly lower on wild potato and their volatiles were repellent to aphid and attractive to parasitoids.

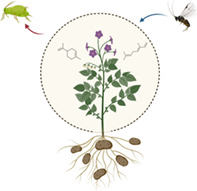

## 1 INTRODUCTION

Current agricultural crops have been selected over a long domestication process which has drastically reduced genetic diversity in crop plants compared to their wild ancestors.^1^, ^2^ Consequently, plant resistance to herbivorous insects can be reduced by crop domestication.^3^, ^4^ Although a meta‐analysis has shown that domestication had no consistent overall effect on the specific plant defence traits underlying resistance, such as secondary metabolites,^4^ with resistance sometimes increased and sometimes decreasing there is still an opportunity to source resistance traits from crop wild relatives. Evidence is available from some examples that show that domesticated plants possess weakened chemical defences compared to their wild ancestor.^5^, ^6^, ^7^

Genetic resources for resistance against insects are limited within commercial cultivated potatoes (*Solanum tuberosum* L.)., resulting in high susceptibility to insect attack.^8^ In contrast, wild potato ancestors grow in a wide range of environmental conditions which render them genetically more diverse than the domesticated and commercially available cultivars of *Solanum* species.^9^ This means that wild potato ancestors could be a potential source of resistant traits that can be utilized in plant breeding programmes.^10^ Supporting this approach, secondary metabolites that play a significant role in plant resistance against insects can be crossed to progenies during crop breeding.^11^ Exploiting potential resistance traits that are available in wild potato ancestors against insects is understudied, as most published work so far has focused on cereal crops.^6^, ^12^ However, there have been some investigations in potato (e.g. Alvarez *et al*.,^13^ Fréchette *et al*.^14^).

Aphids are deleterious pests of many crops that cause yield losses worldwide.^15^ Several species of aphids frequently infest potatoes, causing direct damage (i.e. yield loss from feeding) and indirect damage (i.e. vector plant viruses).^16^ To locate their host, aphids utilize a wide range of cues including visual stimuli [colour, ultraviolet (UV) reflectance spectra], and olfactory stimuli [release of volatile organic compounds (VOCs) from host plants and pheromones from conspecifics].^17^ It is well documented that aphids make use of plant released VOCs and respond accordingly.^18^ More recently, evidence is accumulating about the significant role of plant volatiles in determining the identity and suitability of host plants to aphids and how highly‐specific blends of these volatiles are required to elicit behavioural responses.^19^, ^20^ Furthermore, VOC emission from both aphid‐infested and uninfested leaves allows parasitoids to discriminate between host and non‐host aphid species.^21^, ^22^ As mentioned earlier, wild ancestors of modern crop plants are often more resistant compared to the cultivated plants. Specifically, wild potato negatively affects aphid performance^13^, ^23^ and behaviour.^24^, ^25^

In the present study, we aimed to identify the insect resistance level of wild potato (*Solanum stoloniferum* Schltdl.) accessions compared to cultivated potato (*S. tuberosum)*. To this end, we hypothesized that wild potato accessions better respond to aphid herbivory than commercial potato cultivars. To test this hypothesis, we performed growth assays using peach potato aphids, *Myzus persicae* (Sulzer), as a model herbivore, observing their reproduction and survival on wild and cultivated potato. *Myzus persicae* is one of the most deleterious polyphagous aphids that can damage plants directly through feeding and honeydew deposits, or indirectly by transmitting plant viruses.^16^ In addition to aphid performance and survival studies, the behavioural response of *M. persicae* and its common endoparasitoid [*Diaeretiella rapae* (M'Intosh)] was investigated in an olfactometer bioassay. To correlate insect behavioural response with the emitted VOC profiles, volatile entrainment was conducted and the collected VOCs from wild and cultivated potatoes were analysed and compared.

The outcomes of our study could provide further insights into the potential of using wild potato ancestors as a new source of aphid resistance that can be exploited to optimize the breeding programmes of cultivated potato.

## 2 MATERIALS AND METHODS

### 2.1 Plants

Wild potato, *S. stoloniferum* Schltdl., seeds of the genetic lines (CGN18333, CGN22718, CGN23072) used in experiments were obtained from the Wageningen Centre for Genetic Resources (CGN), Wageningen University, The Netherlands, while *S. tuberosum Desirée* tubers (grown by Nick Crane, Norfolk, UK) were purchased from Sainsbury's supermarket, UK. All plants were grown under controlled environment conditions [20 °C, 37% relative humidity (RH), 16 h:8 h photoperiod] in a growth chamber (MLR‐352‐PE, Panasonic, The Netherlands). Potato plants were grown individually in 7.5 cm pots in John Innes No. 2 compost (Westland Horticulture Ltd, Tyrone, UK). Four‐five week old plants were used for the experiments.

### 2.2 Insects

*Myzus persicae* aphids, originally obtained from Harper Adams University (Newport, UK), were reared in the insectary in the Centre of Applied Entomology and Parasitology (CAEP) at Keele University, UK. *Myzus persicae* clone O was reared on Pak choi *Brassica chinensis*, commonly known as Chinese cabbage, in Bugdorm cages (46 cm × 46 cm × 46 cm; NHBS Ltd, Totnes, UK) under controlled conditions (24 °C, 38% RH, 16 h:8 h photoperiod). The aphid parasitoid *D. rapae* (obtained from Harper Adams University) was reared on Pak choi plants infested with *M. persicae*. To rear parasitoids, mummies of *D. rapae*, attached to plant leaves, were introduced to cages containing fresh Pak choi plants infested with *M. persicae* and kept under controlled condition (20 °C, 40% RH, 16 h:8 h photoperiod). Upon emergence, parasitoid adults were provided with honey solution (1:1 in water) as food. Only female parasitoids were used in experiments and they were 2–3 day old and mated.

### 2.3 Aphid performance bioassay

Performance of *M. persicae* was assessed on the wild and cultivated potato species. There were two separate series of experiments with different plants; the first series recorded observations after 48 h and the second recorded observations after 96 h. Fresh plants and aphids were used in both observations in each experiment. In each replicate, ten adult alate *M. persicae* were placed in a clip cage (2.5 cm diameter; BioQuip Product Inc., Rancho Dominguez, CA, USA), which was attached to the lower surface of plant leaves as described in Sobhy *et al*.^26^ Two clip cages were placed on each plant. Ten replicates were performed for each accession. To assess the survival and fecundity of aphids, plants containing clip cages were left undisturbed in a controlled environment room (25 °C, 37% RH, 16 h:8 h photoperiod). Plants were assessed after the 48 h (series 1) and 96 h (series 2) period. For assessment, leaves containing the cages were cut and cages were removed without losing any aphids. Number of live adults and nymphs produced were recorded.

### 2.4 Volatile collection

Plants volatiles were collected following the procedure described by Ali *et al*.,^27^ which allows collection of plant volatiles in a similar ratio as naturally produced. Plants were placed inside oven bags (25 cm × 38 cm; Bacofoil, Telford, Shropshire, UK, flower seal roasting bags). To avoid any contamination that may come from manufacturing material, bags were baked in an oven (Heraeus, Thermo Electron corporation, Mark Biosciences, UK) at 120 °C overnight prior to entrainments. Porapak Q adsorbent filters (0.05 g, 60/80 mesh; Supelco, Bellefonte, PA, USA) were washed with diethyl ether and then conditioned before use. Plants were enclosed in bags individually. Each bag was open at the bottom and closed at the top. An outlet hole was made in the upper part of the bag to connect the Porapak Q filter, whereas the base bottom was closed by attaching a rubber band around the pot. Charcoal filtered air was pumped into bags at 600 mL/min and sampled air was pulled out at 400 mL/min through the Porapak Q filter in which the plant volatiles were trapped. To avoid the entry of unfiltered air, positive pressure was created by maintaining the difference in flows rates of air in and air out. Connections were made with 1.6 mm inner diameter (i.d.) polytetrafluorethylene (PTFE) tubing (Alltech Associates Inc., Carnforth, UK) with Swagelok brass ferrules and fitting (North London Valve Co., London, UK) and sealed with PTFE tape (Gibbs & Dandy Ltd., Luton, UK). Volatile collection was done for a 48 h period, after which the Porapak filters were eluted with 500 μL of diethyl ether into the sample vials [Supelco, 2 mL PTFE/silicone, (bonded) 9 mm] and stored at −20 °C in a freezer (Lec Medical, UK) for chemical analysis or olfactometer bioassay.

### 2.5 Aphid and parasitoid olfactometer bioassay

The behavioural response of alate *M. persicae* and *D. rapae* to potato VOCs was investigated using a perspex four‐arm olfactometer in a controlled environment room (24 °C, 30 ± 2% RH) as described in Ali *et al*.^27^ At the top of the olfactometer, the central area contained a hole into which a single alate *M. persicae* or female *D. rapae* was introduced, which was connected to a low‐pressure air pump. Air was pulled from the centre of the olfactometer by a vacuum pump with a layer of muslin to prevent access by the tested insect during the bioassays. All replicates were carried out under uniform illumination. The olfactometer arena was split into five areas; four areas by each arm (one VOC treatment and three solvent control arms) and a central area.^28^ Each replicate was run for 12 min, and after every 3 min, the position of the olfactometer was rotated clockwise by 90° to eliminate any directional bias.^27^ Time spent by the insect in each arm was recorded using a software program (OLFA, F. Nazzi Udine, Italy). Ten replicates were done for each insect. Filter paper (110 mm diameter; Whatman filter paper) strips (cut to 5 mm × 20 mm) were treated with an aliquot (10 μL) of the test solution, applied using a micropipette (Drummond ‘microcaps’; Drummond Scientific Co., Broomall, PA, USA). One arm was assigned to the collected VOCs from the potato plants (wild potato lines or Desirée), whereas three control vessels were treated similarly with the same volume of solvent (diethyl ether) on the filter paper strips. If an insect remained motionless for the first 2 min of a replicate, that replicate was discarded. All bioassays were performed between 10:00 and 13:00.

### 2.6 Volatile analysis

Volatile analysis was carried out on a 7820A GC coupled to a 5977B single quad mass selective detector (Agilent Technologies, Cheadle, UK). The gas chromatograph was fitted with a non‐polar HP5‐MS capillary column (30 m × 0.25 mm × 0.25 μm film thickness) coated with (5%‐phenyl)‐methylpolysiloxane (Agilent Technologies) and used hydrogen carrier gas at a constant flow rate of 1.2 mL min^−1^. Automated injections of 1 μL were made using a G4513A autosampler (Agilent Technologies) in splitless mode (285 °C), with oven temperature programmed from 35 °C for 5 min then at 10°C min^−1^ to 285 °C. Compounds were identified according to their mass spectrum, linear retention index relative to retention times of n‐alkanes, and co‐chromatography with authentic compounds.

### 2.7 Statistical analysis

#### 2.7.1 Aphid clip cage bioassay

Differences in the mean number of live adult aphids and produced nymphs on wild (CGN18333, CGN22718, CGN23072) and cultivated (Desiree) potato plants were compared at two time‐points (48 and 96 h) by one‐way analysis of variance (ANOVA). Prior to analysis, data were examined for a normal distribution using the Shapiro–Wilk test. Comparisons among means were performed using Holm–Sidak method (*P* < 0.05).

#### 2.7.2 Olfactometer bioassay

Data on the behavioural response of *M. persicae* and *D. rapae* were analysed by a paired *t*‐test (one tail). In this analysis, the time spent by the tested individuals in treated and the average of three control arms in the four‐arm olfactometer were compared.^29^

#### 2.7.3 Volatile profiling

To visualize the overall differences in volatile profiles emitted from wild (CGN18333, CGN22718, CGN23072) and cultivated (Desiree) potato plants, a principal component analysis (PCA) was performed using the concentrations of the detected volatiles as dependent variables. Loading and score plots were derived after mean‐centring and log transformation of volatile data. VOC visualization was done using the MetaboAnalyst online tool suite.^30^ Subsequently, univariate analysis (*F*‐test) of variances was performed to investigate whether the concentrations of individual volatile compounds differed between wild and cultivated potato plants. All univariate analyses were performed using SigmaPlot 12.3 (Systat Software Inc., San Jose, CA, USA).

## 3 RESULTS

### 3.1 Aphid performance

**Figure 1:**
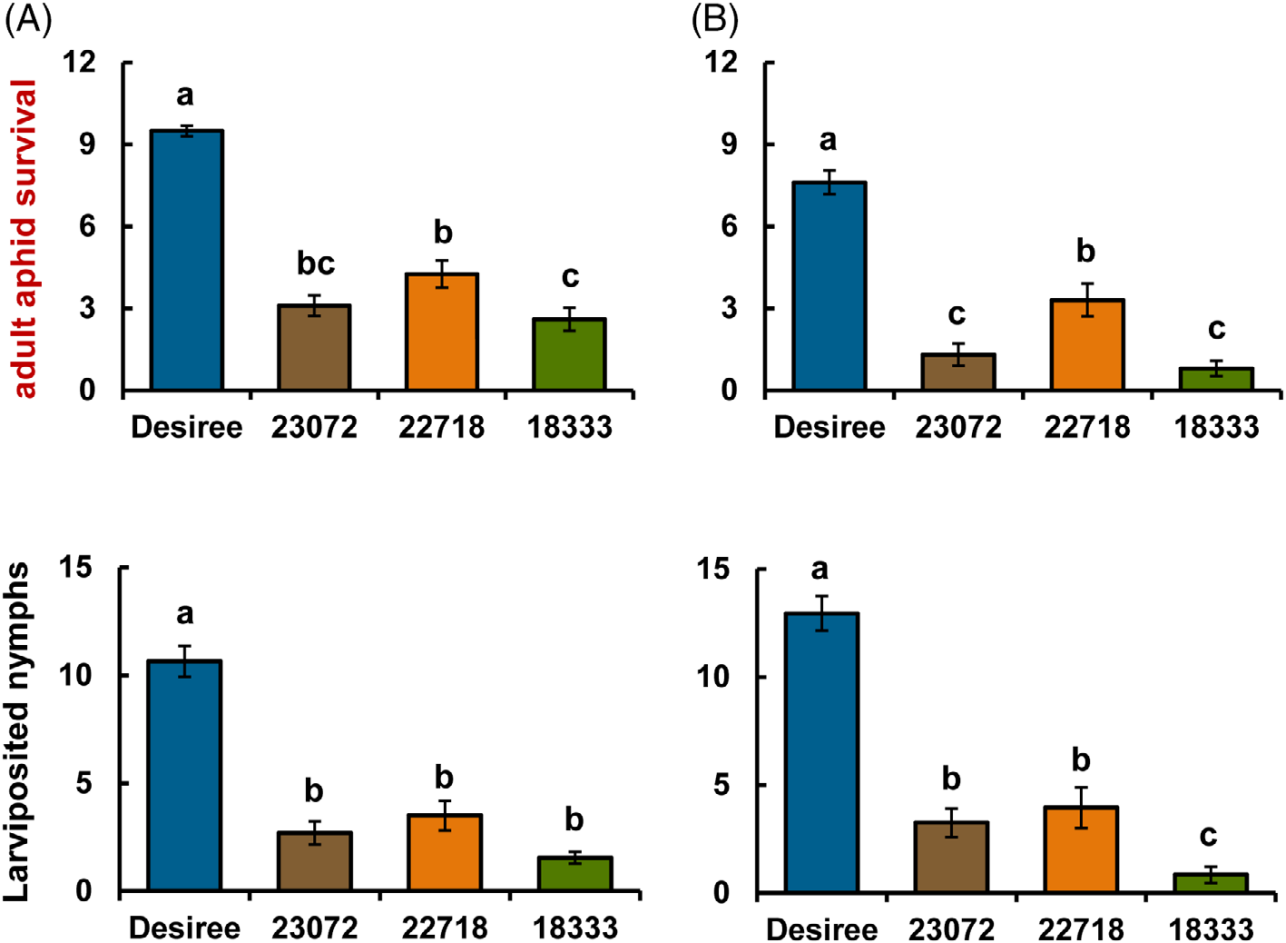
Performance of *Myzus persicae* on cultivated (*Solanum tuberosum*. cv. *Desiree*) and wild (*Solanum stoloniferum*) potato lines. Mean number (± standard error) of surviving adult aphids and larviposited nymphs of *M. persicae* after 48 h (A) and 96 h (B). Different letters indicate statistically significant differences among plant species (*F*‐test; *P* < 0.05), based on the Holm–Sidak method (one‐way ANOVA).

**Figure 2:**
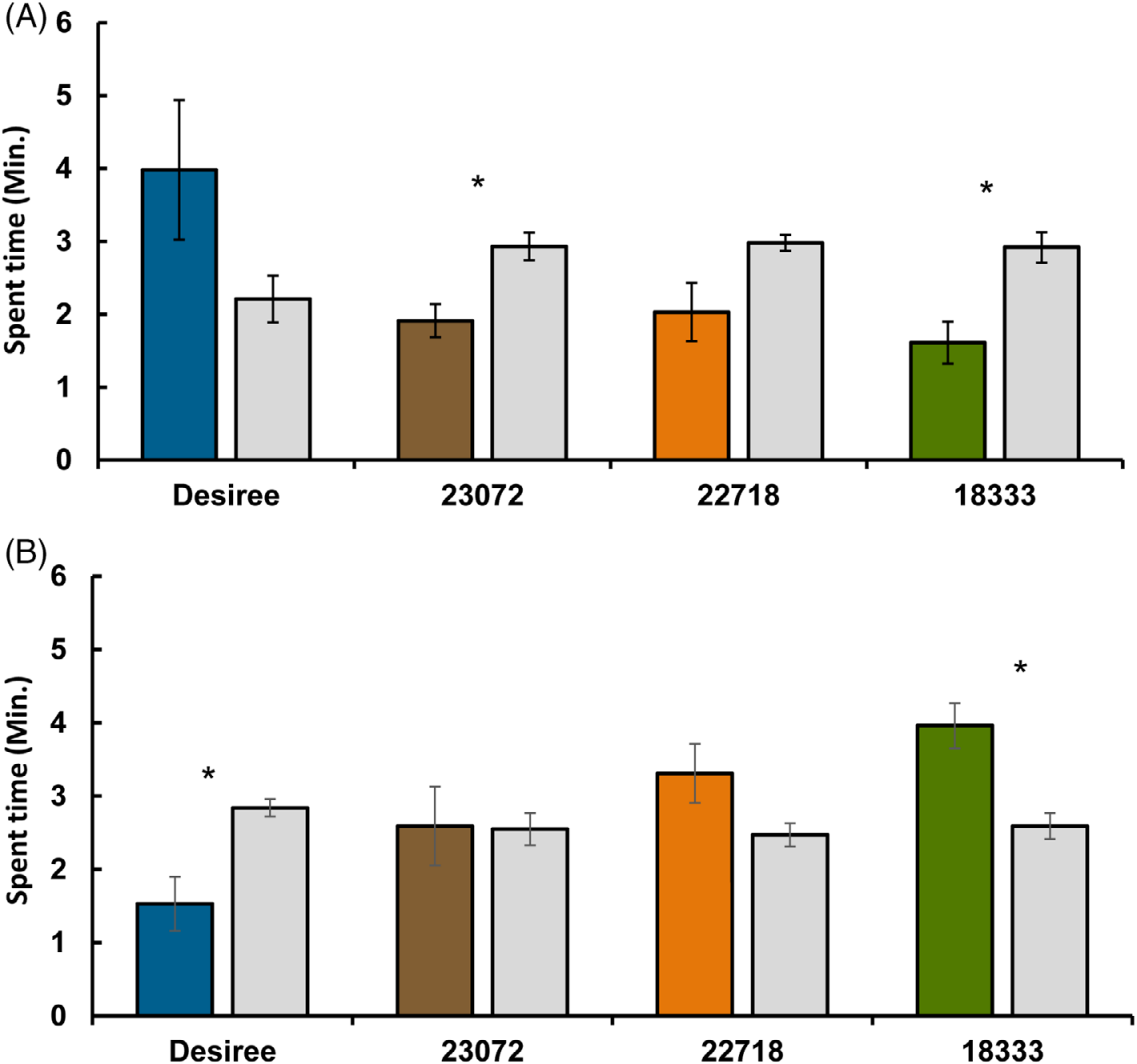
Behavioural responses of *Myzus persicae* (A) and *Diaeretiella rapae* (B) to headspace volatiles from cultivated (*Solanum tuberosum*. cv. *Desiree*) and wild (*Solanum stoloniferum*) potato lines in a four‐arm olfactometer bioassay. Each insect was given 12 min to make a choice between one arm of plant volatiles (coloured bars) *versus* three solvent diethyl ether (DEE) arms (grey bars). The values shown are mean time spent in the arm ± standard error (*n* = 10). Asterisks (*0.01 ≤ *P* ≤ 0.05) above bars indicate statistically significant differences based on a paired *t*‐test (one tail).

After 48 h, there was a significant reduction in the number of adult *M. persicae* surviving on wild potato accessions (Fig. 1) in clip cage experiments. The number of live adult *M. persicae* on Desiree was significantly higher, up to more than three‐fold, compared to the number on wild plants (*F*_3,76_ = 63.732; *P* < 0.001; Fig. 2(A) ). A similar pattern was observed in clip cage experiments after 96 h (Fig. 1(B) ). All wild accessions had significantly higher aphid mortality with less than 35% rate of survival (*F*_3,76_ = 46.299; *P* < 0.001; Fig. 2(A)). Accessions CGN18333, CGN23072 and CGN22718 had 8%, 13% and 33% survival of aphids, respectively. In contrast, Desiree had the least mortality with more than 75% survival rate after 96 h.

There was a significant reduction in nymph production on wild potato accessions across both time points compared to cultivated potato (Fig. 1(A,B)). Mean number of nymphs produced was significantly lower on wild accessions after 48 h (*F*_3,76_ = 48.428; *P* < 0.001), particularly on CGN18333 and CGN22718. The same was also true after 96 h (*F*_3,76_ = 50.739; *P* < 0.001).

### 3.2 Aphid and parasitoid olfactometer bioassay

In an olfactometer bioassay, *M. persicae* were repelled by the volatiles of wild accessions, which was not the case for Desiree (*P* = 0.19). In particular, the accessions CGN18333 and CGN23072 had a significant repellent effect on *M. persicae* with *P* values of 0.013 and 0.018, respectively (Fig. 2(A)).

In contrast to *M. persicae*, volatiles collected from wild accessions attracted parasitoids and volatiles collected from Desiree repelled the parasitoid (Fig. 2(B)). Accession CGN18333 was the only wild potato accession that had a significant attractant effect on *D. rapae (P* = 0.012).

### 3.3 Plant volatile profiles

**Figure 3:**
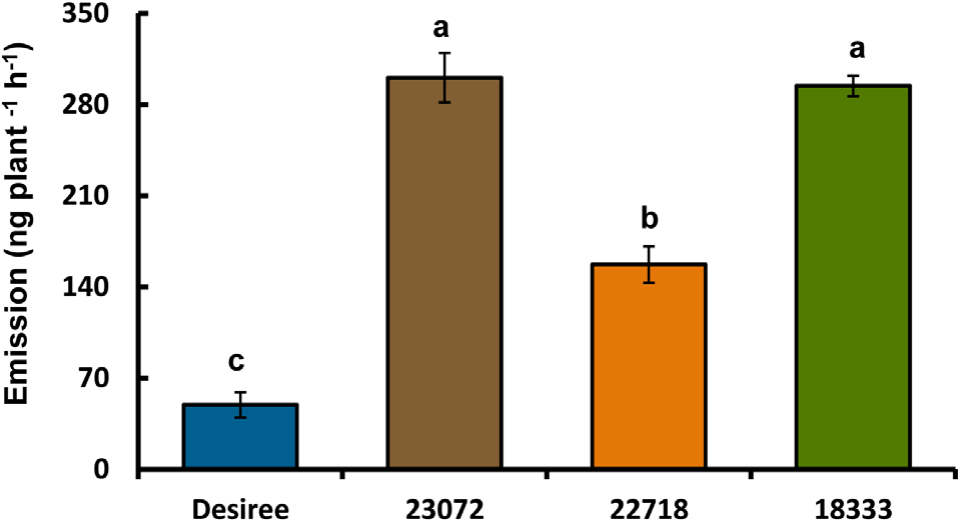
Total amounts (mean ng plant^−1^ h^−1^ ± standard error) of identified volatile organic compounds (VOCs) emitted from cultivated (*Solanum tuberosum*. cv. *Desiree*) and wild (*Solanum stoloniferum*) potato lines. Different letters indicate statistically significant differences among plant species (*F*‐test; *P* < 0.05), based on the Holm–Sidak method (one‐way ANOVA).

Gas chromatography–mass spectrometry (GC–MS) analysis of headspace collections from wild (CGN18333, CGN22718, CGN23072) and cultivated (Desiree) potato plants revealed 23 detectable VOCs in seven functional classes (alcohols, aldehydes, benzenoids, ketones, monoterpenes, homoterpenes and sesquiterpenes; Table 1). There were significant quantitative changes, with a 3–7‐fold increase in the total emitted volatiles of wild accessions compared to Desiree plants (*F*_3,12_ = 61.20; *P* < 0.001; Fig. 3). In addition, most volatile compounds in the earlier‐mentioned VOC groups were emitted from wild accessions in significantly higher amounts compared to Desiree plants (Table 1).

**Table 1:** Emission (in ng per plant^−1^ h^−1^; mean ± standard error; *n* = 3) of volatiles released by cultivated (*Solanum tuberosum.* cv. Desiree) and wild (*Solanum stoloniferum*) potatoes lines

Plant volatiles	KI	*Solanum stoloniferum*			*Solanum tuberosum*	*P* Value
		CGN23072	CGN22718	CGN18333	Desiree	
*Alcohols*						
Phenylethyl alcohol	1116	3.89 ± 1.26	4.25 ± 0.87	2.63 ± 0.58	2.59 ± 0.82	0.688
*p*‐Cymen‐7‐ol	1289	119.27 ± 11.45^a^	17.52 ± 6.48^c^	64.32 ± 19.07^b^	1.37 ± 0.38^d^	**<0.001**
*Aldehydes*						
4‐Ethyl‐benzaldehyde	1122	63.49 ± 8.63^a^	7.31 ± 3.19^b^	21.60 ± 6.47^b^	1.11 ± 0.10^b^	**<0.001**
*Benzenoids*						
MeSA	1192	7.81 ± 0.93^b^	26.08 ± 6.08^a^	4.39 ± 0.51^b^	3.25 ± 0.37^b^	**0.002**
Benzothiazole	1229	1.05 ± 0.52^b^	7.59 ± 1.42^a^	10.07 ± 0.93^a^	1.00 ± 0.09^b^	**0.002**
*Ketones*						
MHO	989	13.15 ± 0.65	4.39 ± 1.66	10.69 ± 2.79	0.49 ± 0.24	**0.024**
*Monoterpenes*						
β‐Myrcene	992	3.65 ± 0.41	5.95 ± 1.38	3.75 ± 0.42	1.84 ± 0.61	0.062
*p*‐Cymene	1026	1.79 ± 0.14	1.86 ± 0.20	1.78 ± 0.17	2.56 ± 0.19	0.104
d‐Limonene	1030	20.39 ± 2.09^a^	23.99 ± 2.86^a^	19.83 ± 1.98^a^	0.71 ± 0.25^b^	**0.003**
Linalool	1099	8.71 ± 2.76^a^	9.37 ± 1.58^a^	8.83 ± 0.60^a^	2.52 ± 0.76^b^	0.083
*Homoterpenes*						
DMNT	1116	0.76 ± 0.08^b^	2.15 ± 0.18^b^	1.15 ± 0.22^b^	11.26 ± 3.24^a^	**0.045**
TMTT	1577	2.62 ± 0.38^b^	4.10 ± 0.84^b^	23.68 ± 3.89^a^	0.79 ± 0.08^b^	**<0.001**
*Sesquiterpenes*						
β‐Cubebene	1351	10.27 ± 2.46	5.62 ± 2.17	4.09 ± 0.86	7.38 ± 3.86	0.497
α‐Copaene	1376	3.70 ± 0.58^c^	7.34 ± 0.64^b^	15.12 ± 1.08^a^	1.89 ± 0.59^c^	**<0.001**
β‐Elemene	1391	4.07 ± 1.67^b^	2.67 ± 0.18^b^	40.52 ± 14.16^a^	1.02 ± 0.04^b^	**0.014**
Longifolene	1402	3.29 ± 0.72	2.72 ± 0.76	2.72 ± 0.69	ND	0.858
Caryophyllene	1419	5.49 ± 0.67	6.89 ± 0.59	4.72 ± 0.28	7.92 ± 2.47	0.485
*trans*‐α‐Bergamotene	1435	1.35 ± 0.14^b^	3.54 ± 0.32^b^	20.69 ± 7.69^a^	0.81 ± 0.08^b^	**0.024**
(*E*)‐β‐Farnesene	1457	1.61 ± 0.11^b^	4.34 ± 0.38^ab^	10.13 ± 3.68^a^	1.31 ± 0.13^b^	**0.045**
Germacrene D	1481	21.23 ± 5.45^a^	1.49 ± 0.08^c^	6.68 ± 1.70^b^	1.25 ± 0.21^c^	**0.005**
β‐Selinene	1486	1.41 ± 0.09^b^	6.31 ± 1.21^a^	7.27 ± 0.63^a^	0.97 ± 0.16^b^	**<0.001**
β‐Bisabolene	1509	1.59 ± 0.20^c^	2.78 ± 0.26^b^	5.07 ± 0.45^a^	0.83 ± 0.05^c^	**<0.001**
Nerolidol	1566	1.68 ± 0.05	2.11 ± 0.07	6.49 ± 2.71	0.87 ± 0.15	0.101
Total emitted volatiles		300.68 ± 18.48^a^	157.19 ± 13.89^b^	294.32 ± 7.91^a^	49.42 ± 9.76^c^	**<0.001**

Under each chemical class, volatile organic compounds (VOCs) are ordered in accordance with their increasing retention time in a gas chromatograph and Kovats index (KI). DMNT, (*E*)‐4,8‐dimethyl‐1,3,7‐nonatriene; TMTT, (*E,E*)‐4,8,12‐trimethyl‐1,3,7,11‐tridecatetraene; MeSA, methyl salicylate; MHO, 6‐methyl‐5‐hepten‐2‐one; ND, not detected. VOCs were tentatively identified based on spectra, Kovats retention index and NIST 17 library matches. KI, Kovats index determined on the intermediately non‐polar HP5‐MS column (https://webbook.nist.gov/; http://www.pherobase.com/). Different letters in the same row indicate significant differences between potato lines (one way analysis of variance; *P* < 0.05). The *P*‐values in bold indicate significant difference.

**Figure 4:**
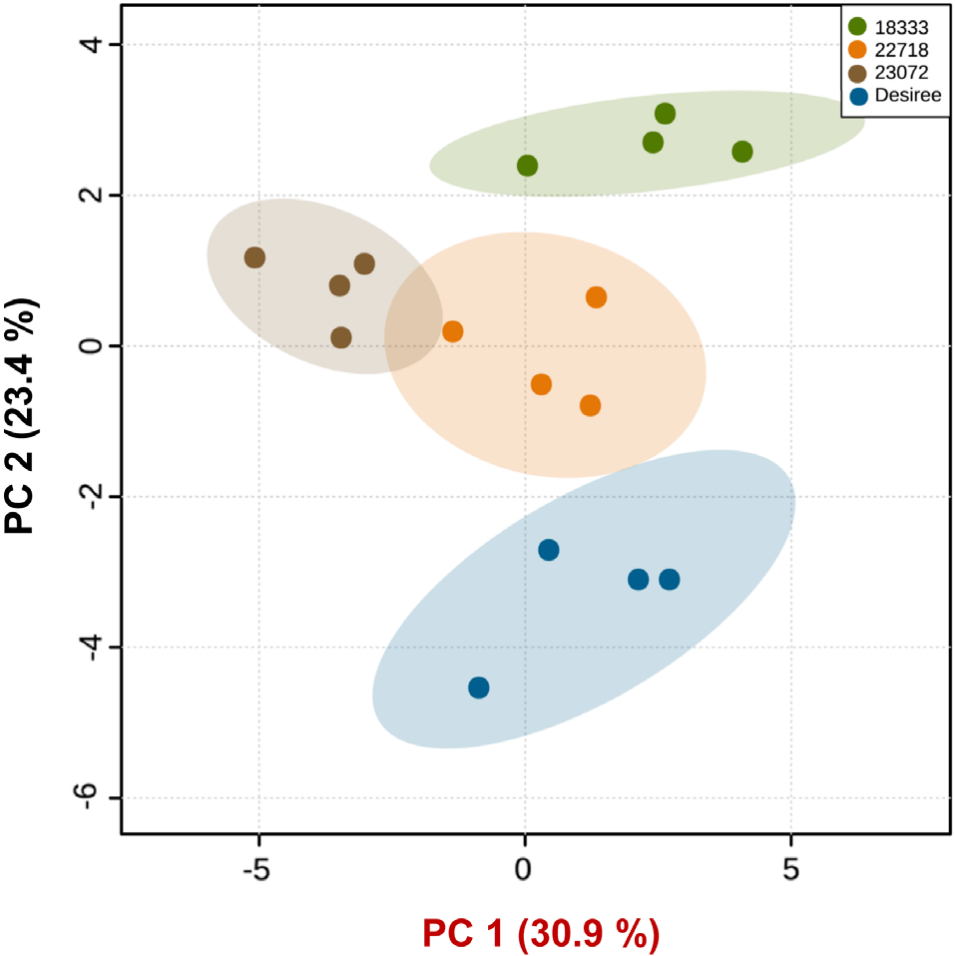
PCA of volatile compounds emitted by cultivated (*Solanum tuberosum*. cv. *Desiree*) and wild (*Solanum stoloniferum*) potato lines (*n* = 4) sampled for 48 h. The score plot visualizes the ordination of collected samples according to the first two PCs based on the quantity of different volatiles emitted from different potato lines, with the percentage of the variation explained in parentheses. The ellipses show 95% confidence regions.

PCA of the VOCs showed that the first two principal components (PCs) accounted for 54.3% of the total variation in the volatile data (Fig. 4). Hence, these two PCs illustrated most of the variation in the data of likely biological relevance. A clear separation based on the second principal component (PC2) is visible between the volatile profiles of wild (CGN18333, CGN22718, CGN23072) in one cluster and cultivated (Desiree) potato plants, whereas another separation but based on the first principal component (PC1) is obvious for the volatile profiles of CGN23072 and a cluster of CGN18333, CGN22718 and Desiree plants. The greatest loadings of PC2, in descending order, were for d‐limonene (0.285), (*E,E*)‐4,8,12‐trimethyl‐1,3,7,11‐tridecatetraene (TMTT) (0.272), and *p*‐cymen‐7‐ol (0.255), whereas the major loadings of PC1 were for β‐bisabolene (0.293), (*E*)‐β‐farnesene (0.288), and *trans*‐α‐bergamotene (0.285). This suggests that these VOCs, shown to contribute to PC1 and PC2, may impact the behaviour response of both *M. persicae* and *D. rapae*.

## 4 DISCUSSION

The current study found evidence of aphid, *M. persicae*, resistance in wild potato, *S. stoloniferum*, accessions. Aphid survival and performance were significantly lower on wild potato accessions compared to cultivated potatoes and volatiles from the wild accessions were also repellent. Volatile analysis revealed that wild accessions released higher amounts of volatile compounds compared to cultivated potatoes.

There was a significant difference in the susceptibility of wild and cultivated species of *Solanum*. The most resistant accession of wild potato, *S. stoloniferum*, in this experiment, was CGN18333, followed by CGN23072 and CGN22718. The cultivated potato, *S. tuberosum*, variety Desiree was found to be susceptible and had substantial nymph production after 96 h. Nymph production was reduced in wild accessions and there was high mortality of adults. In all three *S. stoloniferum* accessions, a significant number of adults was dead after 96 h; survival was less than 10% on accessions CGN18333 and CGN23072. Such reduced performance of *M. persicae* was also reported for other wild *Solanum* species (i.e. *S. trifidum*, *S. polyadenium*, *S. tarijense* and *S. palustre*). Of these previously reported species, *S. palustre* was the most resistant to *M. persicae* as it contained the highest number of glandular hairs, which is one of the aphid resistance factors in *Solanum*.^14^, ^31^ The same was also true for the potato aphid (*Macrosiphum euphorbiae* Thomas) on wild *Solanum berthaulti*.^32^ Such reduced aphid survival on wild potato could be also attributed to the high content of alkaloids that is commonly found in wild potato, which could be responsible for antibiotic effects observed on aphids.^33^, ^34^

In olfactometer experiments, aphids were repelled by the odours of accessions CGN18333 and CGN23072. Similarly, the wild potato *S. berthaultii* was very repellent to *M. persicae* ^24^, ^35^ but this was attributed to burst emission of the aphid alarm pheromone, (*E*)‐β‐farnesene, from trichomes. In our study a mixture of volatiles was released by *S. stoloniferum*. Conversely, the olfactometer studies showed that wild accessions also help in recruiting natural enemies as the parasitoid *D. rapae* spent more time in arm treated with volatiles from wild plants of accession CGN18333. In contrast, parasitoids spent significantly less time on Desiree plants. Chemical analyses revealed that wild accessions released higher amounts of volatile compounds compared to cultivated potatoes. Consistent with our findings, it has been shown that commercial cultivars have lost the ability to produce certain key volatiles in response to herbivory, which thereby negatively affected natural enemy recruitment.^36^, ^37^, ^38^, ^39^ In contrast, a recent meta‐analysis by Rowen and Kaplan^40^ showed that domesticated species induced stronger volatile responses to herbivory than wild species. However, they note that their data show that domestication reduces the complexity of volatile blends emitted and thus critical compounds for natural enemy attraction may be missing. There is little published evidence documenting how crop domestication has affected indirect plant defence, therefore, a general conclusion cannot be drawn.^3^ A genome wide association study of 146 maize genotypes comprising of landraces, inbred lines and commercial hybrids found that herbivore egg induced attraction of stemborer parasitoids could be found in some high yielding improved maize lines and commercial hybrids but was more frequent in landraces.^41^

Multivariate analysis of volatile data revealed that β‐bisabolene, (*E*)‐β‐farnesene, *trans*‐α‐bergamotene, d‐limonene, TMTT, and *p*‐cymen‐7‐ol were produced in larger amounts by the wild potato accessions, and it is likely that these compounds played a role in repelling aphids and attracting their natural enemies. Previous work has shown an increased emission of β‐bisabolene,^26^ (*E*)‐β‐farnesene,^42^ *trans*‐α‐bergamotene,^43^ d‐limonene,^44^ TMTT,^42^, ^45^ *p*‐cymen‐7‐ol^44^ and in response to aphid herbivory and potato induction with defence elicitors. This can provide an explanation for the negative response of aphids to wild accessions as aphids may perceive such elevated volatile profiles from wild accessions as signals of a greater risk of competition from conspecifics.^20^ Supporting our findings, TMTT was significantly repellent to *M. persicae* when tested individually.^46^ Similarly, it is well documented that (*E*)‐β‐farnesene functions as an alarm pheromone and also serves as a host finding kairomone for aphid natural enemies.^47^, ^48^, ^49^

Previous studies reported that plant secondary metabolites could provide a way to enhance plant resistance^50^ by reducing the survival and reproductive rate of insect herbivores.^51^ In addition, recruitment of natural enemies is an important component of plant in defence against herbivore attack.^52^ Plants that produce appropriate volatile compounds successfully recruit a wide range of natural enemies.^53^ These plants can contribute to establishing a sustainable agricultural system, by enhancing biological control of pests, but the quantity and quality of plant volatiles released is critical for attraction of natural enemies to plants.^52^ Sometimes compounds present in small quantities are more biologically active despite small quantities.^54^, ^55^ Quality, quantity, and the ratio of volatile compounds all play a crucial role in plant–insect interactions.^56^ Commercially available crop plants have been bred primarily to obtain higher yield. This genetic change through selection for yield may compromise plant defence capacity by altering the interaction between plants, herbivores, and natural enemies if defence traits have a yield penalty.^3^

## 5 CONCLUSION

The current study shows that there are promising sources of direct aphid resistance in the *S. stoloniferum* accessions tested. The very low aphid survival observed suggests that toxic phytochemicals were present. Although the potential of crop wild relatives as sources of novel resistance to insect pests has been extensively studied, the progress of transferring resistance traits from wild cultivars is still slow. This is attributed to difficulties in identifying the key secondary metabolites that determine resistance and the genes encoding their production. Thus, identification of the bioactive compounds and genes encoding resistance will be important topics for future studies. To retain the marketable quality required, it will be also important to test if bioactive compounds are harmful to humans or if they affect the taste of the potatoes. Morphological differences could be seen between the *Solanum* species which were used in experiments; wild accessions had smaller leaves compared to Desiree. Although there could be some relation between aphids and leaf size it is unlikely to explain the high mortality observed in the current study which is more likely due the presence of toxic phytochemicals. The current research findings open up the prospect of breeding for aphid resistance by crossing cultivated and wild potatoes.

## AUTHOR CONTRIBUTIONS

JA, ISS and TJAB conceived the ideas and designed the experiments. JA performed the experiments and collected the data. ISS performed the GC chemical analysis and interpreted the volatile data. JA, ISS and TJAB wrote the manuscript. All authors contributed critically to the drafts and gave final approval for publication.

## CONFLICT OF INTEREST

The authors declare that they have no conflict of interest.

## Data Availability

The data that support the findings of this study are available from the corresponding author upon reasonable request.
